# The Gorlin-Goltz syndrome: a sporadic case

**DOI:** 10.11604/pamj.2014.17.55.3212

**Published:** 2014-01-25

**Authors:** Kawtar Inani, Fatimazahra Mernissi

**Affiliations:** 1 Service de Dermatologie, CHU Hassan II, Fez, Morocco

**Keywords:** Gorlin-Goltz syndrome, basal cell nevus syndrome, hereditary disorder

## Image in medicine

Gorlin-Goltz syndrome, also known as basal cell nevus syndrome, is an uncommon autosomal dominant hereditary disorder; which is characterized by numerous basal cell carcinomas, maxillary keratocysts and bones malformations. It results from a mutation of the PATCHED gene. The estimate incidence for a general population is 1/50000 to 1/150000. We present a 30 year-old woman, with no similar familial case, who had a repeatedly dental abscesses since the age of 15 years, she consulted for papulonodular lesions that have appeared at the age of eighty. Those lesions were localized on the face and the trunk, reminiscent of basal cell carcinomas. The clinical examination found papulonodular lesions, a mandibular fistula, broadened nasal root, palmer pits and a swelling at the right big toe. The dermoscopic examination of the papulonodular lesions showed a kind of trunk tree arborisation. The biopsy excision of the swelling at the right toe and papulonodulor lesions, revealed respectively an epidermoid cyst and basal cell carcinomas. Radiographic examination showed a calcified falx cerebri calcification, bifid ribs, synostosis, wedged, scoliosis and agenesis of the left kidney. Our patient was treated by a surgical excision of skin lesion with a setting flat of a mandibular fistula. Also a photoprotection was recommended and a regular monitoring of the skin lesions. The Evolution was marked by the emergence of new basal cell carcinomas treated by photodynamic therapy.

**Figure 1:**
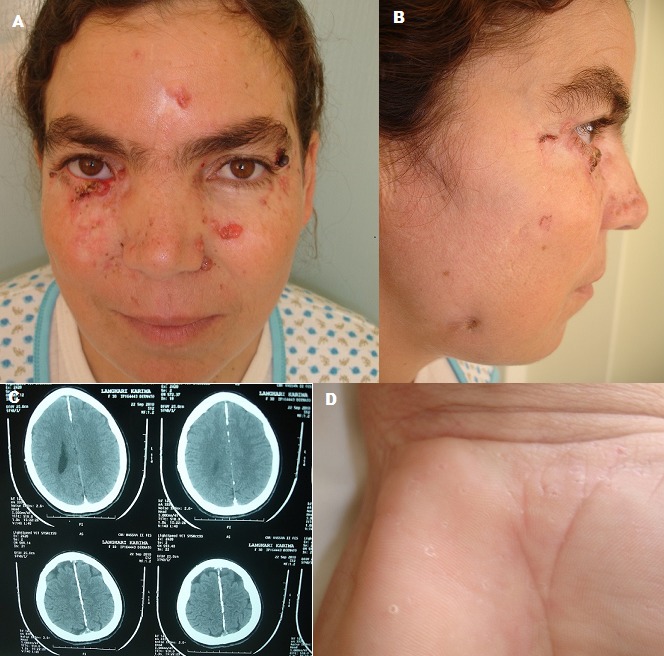
A) papulonodular lesions and a broadened nasal root; B) A mandibular fistula; C)MRI image shows a calcified falx cerebri calcification; D) palmer pits

